# Eating Disorders in Schizophrenia: Implications for Research and Management

**DOI:** 10.1155/2014/791573

**Published:** 2014-11-18

**Authors:** Youssef Kouidrat, Ali Amad, Jean-Daniel Lalau, Gwenole Loas

**Affiliations:** ^1^Department of Endocrinology, University of Picardie-Jules Verne, CHU d'Amiens, Amiens, France; ^2^Department of Nutrition, Hôpital maritime, Assistance Publique-Hôpitaux de Paris (AP-HP), 62600 Berck, France; ^3^Psychiatry Department and SCA-lab, PSYchiC team, Université de Lille, 59100 Lille, France; ^4^Department of Psychiatry, Erasme Hospital, ULB, 1070 Brussels, Belgium

## Abstract

*Objective.* Despite evidence from case series, the comorbidity of eating disorders (EDs) with schizophrenia is poorly understood. This review aimed to assess the epidemiological and clinical characteristics of EDs in schizophrenia patients and to examine whether the management of EDs can be improved. *Methods.* A qualitative review of the published literature was performed using the following terms: “schizophrenia” in association with “eating disorders,” “anorexia nervosa,” “bulimia nervosa,” “binge eating disorder,” or “night eating syndrome.” *Results.* According to our literature review, there is a high prevalence of comorbidity between schizophrenia and EDs. EDs may occur together with or independent of psychotic symptoms in these patients. Binge eating disorders and night eating syndromes are frequently found in patients with schizophrenia, with a prevalence of approximately 10%. Anorexia nervosa seems to affect between 1 and 4% of schizophrenia patients. Psychopathological and neurobiological mechanisms, including effects of antipsychotic drugs, should be more extensively explored. *Conclusions.* The comorbidity of EDs in schizophrenia remains relatively unexplored. The clearest message of this review is the importance of screening for and assessment of comorbid EDs in schizophrenia patients. The management of EDs in schizophrenia requires a multidisciplinary approach to attain maximized health outcomes. For clinical practice, we propose some recommendations regarding patient-centered care.

## 1. Introduction

Schizophrenia is a severe and frequently observed mental illness that affects 1% of the general population. Schizophrenia is characterized by a wide range of symptoms, including positive symptoms (delusions and hallucinations), negative symptoms (social withdrawal, blunted affect), cognitive symptoms (difficulties with memory and attention), and affective dysregulation [1]. Moreover, psychiatric comorbidities are very common among patients with schizophrenia, particularly substance abuse, anxiety, and depressive symptoms [2]. In addition to these psychiatric features, endocrine and cardiometabolic alterations (e.g., type 2 diabetes, obesity, hypertension, and dyslipidemia) are frequently associated with schizophrenia. Indeed, cardiovascular diseases are the leading cause of the high mortality among patients with schizophrenia, which is 2-3 times higher than that of the general population [3, 4].

The etiology of the cardiometabolic disorders in schizophrenia is multifactorial and includes oxidative stress [5], conventional risk factors such as genetic and lifestyle factors, and drug side effects [6]. In addition, as in the general population, eating behaviors and eating disorders (EDs) are crucial in determining the etiology of cardiometabolic disorders in patients with schizophrenia [7]. EDs have been associated with profound physical and psychosocial morbidity and an elevated mortality risk [8, 9].

Although Eugen Bleuler described disturbances in eating behavior as a feature of schizophrenia in the early nineteenth century [10], EDs in schizophrenia remain understudied and poorly understood by health care providers [11, 12]. Indeed, EDs in schizophrenia remain difficult to assess, and schizophrenia patients with EDs usually do not meet all criteria for typical EDs, leading clinicians to consider EDs a secondary problem [13]. Our review focuses on EDs associated with schizophrenia and offers practical methods for their diagnosis and therapeutic management.

For this work, electronic searches were carried out using Medline, ScienceDirect, and Google Scholar for the following terms: “schizophrenia,” “eating disorders,” “anorexia nervosa,” “bulimia nervosa,” “binge eating disorder,” or “night eating syndrome.” The search was limited to articles published in English and French. Youssef Kouidrat and Ali Amad analyzed the studies, including case reports and case series.

## 2. Eating Disorders Associated with Schizophrenia

### 2.1. Anorexia Nervosa

With the recent release of the fifth iteration of the Diagnostic and Statistical Manual (DSM-5), diagnostic criteria for anorexia nervosa (AN) have undergone several changes [14]. AN is defined by persistent restriction of energy intake leading to significantly low body weight, intense fear of gaining weight, or persistent behavior that interferes with weight gain and disturbance in the way one's body weight or shape is experienced. The requirement for amenorrhea has been eliminated in DSM-5 [15]. AN is relatively common among young women with a lifetime prevalence up to 4% and a female to male sex ratio of 10 to 1 [16]. The mental illnesses most commonly associated with AN are major depression, anxiety disorders, and obsessive-compulsive disorders [17, 18]. Several evidences from case series have demonstrated the possibility of comorbidity between AN and schizophrenia with different prevalence rates [19–24].

The frequency of AN in schizophrenia has been approximated to be between 1 and 4% [11, 24]; the comorbidity between these disorders seems to vary with sex, at 0.81% for men and 4.01% for women, as found by Gotestam et al. in a large outpatient Norwegian psychiatric population, using a staff-report questionnaire [25]. Interestingly, men with AN are 3.6 times more likely to present with an associated diagnosis of schizophrenia than women [24].

AN can occur as a symptom on the spectrum of manifestations of schizophrenia, and overlapping symptoms in the psychopathology of schizophrenia and AN (such as distortion of body image and fear of being fat) are frequently observed. AN may precede or follow schizophrenia. For example, many male patients who are diagnosed with AN are found to have schizophrenia several years after the initial diagnosis (sometimes even 6 years later) [23, 26, 27].

Additionally, various clinical features of schizophrenia can lead to anorexia. For example, a depressive disorder can be associated with schizophrenia and lead to losses of appetite and weight. Second, due to paranoid delusions, the patient may believe that their food or drink is being poisoned or contaminated and refuse to eat it. Finally, acoustico-verbal hallucinations can be perceived as ordering a complete food refusal.

### 2.2. Bulimia Nervosa

Bulimia nervosa (BN) is characterized by recurrent episodes of binge eating followed by repeated inappropriate compensatory behaviors to prevent weight gain (such as self-induced vomiting, fasting, or excessive exercise or a misuse of laxatives or other medications) [28]. BN occurs in 1–3% of the population [16]. According to the DSM-5, binge eating and inappropriate compensatory behaviors should both occur, on average, at least once a week for 3 months.

Very little data exist on BN in schizophrenia. Gotestam et al. showed a prevalence of BN with schizophrenia of 0.73% for men and 1.57% for women [25]. In 1997, Deckelman et al. described a case of four young women with BN. In one case, BN clearly preceded the onset of schizophrenia; in the other 3 cases, the bulimic symptoms interacted significantly with the psychotic symptoms at the time of the diagnosis of schizophrenia. These case reports support a model wherein the coexistence of schizophrenia and bulimia may have important clinical implications [29]. Miotto et al. investigated the occurrence of symptoms of psychosis in 112 female patients diagnosed with DSM-IV eating disorders (AN = 61; BN = 51) and in 631 high school girls in the same health district as the patients. Compared with controls, a higher prevalence of symptoms of psychosis has been observed in patients with EDs; however, no cases of comorbid schizophrenia were observed [30]. In conclusion, there are very few studies on BN and schizophrenia, and the hypothesis of an association between these disorders remains to be proven.

### 2.3. Binge Eating Disorder

Binge eating disorder (BED) has recently been recognized as an ED in the DSM-5. BED is characterized by consuming large amounts of food in a short time period, at once a week for 3 months, and is accompanied by a sensation of losing control over eating. The diagnosis of BED must be associated with at least 3 of the following characteristics: (1) eating much more rapidly than normal, (2) eating until feeling uncomfortably full, (3) eating large amounts of food when not feeling physically hungry, (4) eating alone because being embarrassed of how much one is eating, or (5) feeling disgusted with oneself, depressed, or very guilty after overeating [14]. Moreover, binge eating in BED is not associated with the regular use of inappropriate compensatory behaviors [31].

The prevalence of BED in the general population varies between 0.7% [36] and 4.3% [37], and women are affected approximately 1.5 times more often than men [38]. One study evaluated the symptoms of BED among 31 patients with schizophrenia who were mostly overweight or obese (71% with BMI ≥ 25 kg/m^2^). In this group, five patients (16%) met the criteria for BED, including three who reported the onset of signs after treatment with atypical antipsychotics [39]. Recently, Lundgren et al. showed a 6% prevalence of BED among 68 obese patients with schizophrenia and bipolar disorder [34]. These prevalence rates are higher than those found in the general population (approximately 2%). However, we must be cautious in interpreting this result, given the small sample of patients interviewed.

### 2.4. Night Eating Syndrome

First described by Stunkard et al. in 1955 [40], various diagnostic criteria for night eating syndrome (NES) have been published in recent years. NES is currently included in the “Other Specified Feeding or Eating Disorder” category of the DSM-5. It is characterized by an abnormally increased food intake in the evening and nighttime, manifested by consumption of at least 25% of intake after the evening meal, and/or nocturnal awakenings with ingestions at least twice per week [41]. Indeed, sleep disorders, which are common in schizophrenia [42], affect the hormonal regulation of food intake and are associated with metabolic disorders, obesity, and cardiovascular disease [43]. NES affects 1.5% of the general population, and between 8.9 and 27% of obese individuals [44].

Out of 175 patients with schizophrenia, Palmese et al. found an 8% prevalence of NES, which was significantly associated with increased rates of insomnia (44%), as assessed by the Pittsburgh Sleep Quality Index (PSQI) [35]. Very recently, out of 100 patients with schizophrenia or schizoaffective disorders and based on the* Night Eating Questionnaire* (NEQ), 8% met the full criteria for NES, with an additional 8% meeting the partial criteria [45]. In 2006, out of 205 psychiatric outpatients (regardless of BMI), Lundgren et al. found that 12% of patients met the criteria for NES [46]. Patients with NES were more likely to take antipsychotic medications than those without NES (38.8% versus 30.8%, resp., *P* = 0.04). In this sample, obese patients were five times more likely to meet the criteria for NES than nonobese patients. In 2010, the same team evaluated the prevalence of NES in a sample of 68 obese patients with schizophrenia and bipolar disorder. Using the NEQ, the prevalence appeared to be approximately 25% [34]. These studies suggest that patients with schizophrenia present an increased risk of NES.

### 2.5. Other Eating Disorders

The Eating Disorders section of the DSM-5 now includes avoidant/restrictive food intake disorder, pica, and rumination disorder. These 3 disorders were previously listed in the childhood disorders section of the DSM-IV-TR [15]. Small changes were made to the criteria for pica and rumination disorder.

Pica is defined as the repeated ingestion of nonnutritive substances (pebbles, hair, small metal objects, etc.). This disorder is common in children (and is found more rarely in adulthood) with developmental disorders (e.g., autism) or mental retardation [47]. In schizophrenia, it can be defined as an impulsive consumption associated with delusions. Domingo-Claros et al. reported a case of a 29-year-old woman with schizophrenia and severe anemia who was diagnosed with lead poisoning (saturnism) as a result the pica ingestion of small metal jewelry found in her stomach during an endoscopy [48]. Many cases of coprophagia, defined as the ingestion of feces and considered a variant of pica, have been associated with schizophrenia [49]. Finally, many studies suggest a significant association between schizophrenia and potomania, defined as the ingestion of beverages in large quantities, on the order of 8 to 10 liters per day. In cases of water intoxication, severe metabolic imbalances can occur, leading to hyponatremia, convulsions, and coma [50].

In Table 1, descriptive epidemiological studies conducted using widely used scales in the psychiatric population are summarized.

## 3. Antipsychotics and Eating Behaviors

Antipsychotic drugs remain essential in the therapeutic management of schizophrenia. These treatments have remarkable therapeutic efficacy and result in great improvements in positive symptoms, prevention of deterioration, cognitive function, quality of life, and reduction in the number of (re)hospitalizations [51, 52]. However, they are likely to be associated with varying degrees of metabolic adverse effects, such as weight gain, dyslipidemia, and impaired glucose metabolism. These detrimental metabolic effects are associated with the vast majority of first- and second-generation antipsychotics [53, 54]. Most studies have focused on the importance of weight gain during treatment, and several potential mechanisms have been widely studied. However, the factors related to changes in eating behavior induced by antipsychotics remain surprisingly poorly studied, while changes in food intake and the modification of the signals of satiety have been observed and proposed as mechanisms of weight gain [55].

Very few studies were found concerning food intake modifications associated with antipsychotics. A study evaluating the eating behaviors of 153 patients with schizophrenia using the Three-Factor Eating Questionnaire (TFEQ) and the Dutch Eating Behavior Questionnaire (DEBQ) observed that patients treated with atypical antipsychotics were more responsive to external food cues and had higher scores on the item “loss of control eating behavior” than patients taking first-generation antipsychotics or control subjects [56]. In addition, treatment with clozapine and olanzapine was significantly associated with compulsive overeating and BED [32, 33].

Several mechanisms of weight gain and increased food intake associated with antipsychotics have been proposed and are summarized in the following:

1. direct effects on antipsychotics receptors,
2. direct or indirect effects on the neuronal circuits (hypothalamus) controlling food intake and satiety,
3. disruption of the hypothalamic-pituitary-adrenal axis,
4. direct effect on insulin sensitivity and insulin secretion,
5. effects on gastrointestinal hormones involved in food intake,
6. decreased physical activity and decreased basal metabolism.

Antipsychotic drugs can affect multiple neurotransmitter systems and exert antagonistic actions on dopamine receptors, serotonergics, histamine, muscarinics, and adrenergics [57]. All of these neurotransmitters have been directly or indirectly involved in the pathways associated with the regulation of food intake [58], metabolism [59, 60], and weight balance [61, 62]. Blockades of dopamine (D2 and D3), serotonin (5HT2c), histamine (H1) [63], and muscarinic (M2 and M3) receptors have all been shown to increase appetite [64, 65]. Additionally, by endocrine/metabolic mechanisms, antipsychotics can directly induce the activation of the hypothalamus-pituitary-adrenal axis [66], deficits in insulin secretion [67], and changes in gastrointestinal hormones [68]. Other studies suggested a relationship between the increased food intake induced by an antipsychotic drug and changes in leptin, melatonin, opioid, and endocannabinoid signaling [69]. Moreover, the weight gain liabilities of antipsychotic drugs seem to be partly associated with their ability to increase appetite [70].

Despite the relevance of these studies, it is clear that the mechanisms underlying the effects of antipsychotics on eating behavior are insufficiently understood. A better understanding of the role of changes in eating behaviors during antipsychotic treatment is necessary for both clinicians and patients. Indeed, any change in appetite could be a harbinger of weight gain, and, thus, preventive measures (choice of antipsychotic treatment, patient education and/or counseling on lifestyle and dietary rules) with a demonstrated efficacy should be taken [71]. In addition, patients and their families should be informed in advance of these side effects so that they are better able to manage them.

## 4. Discussion and Recommendations

In clinical practice, many combinations of EDs and schizophrenia are possible, as these diagnoses are certainly not mutually exclusive. The EDs may occur together with or independent of the psychotic symptoms of patients [13]. In some clinical situations, ED coexists as a separate clinical entity of schizophrenia. Sometimes, the ED represents a symptom that may be the first manifestation of a psychotic disorder, such as schizophrenia.

However, despite a high prevalence of comorbidity between schizophrenia and EDs, this topic remains relatively unexplored. According to our literature review, the prevalence of EDs differed according to the methodology, sample sizes, and scales of the studies. Thus, AN affects between 1 and 4% of patients with schizophrenia. In addition, BED and NES have an average prevalence of 5–20% in the schizophrenia population (approximately 5 times higher than in the general population) [34, 45].

The origin of the development of EDs in these patients remains unclear, and related psychopathological and neurobiological mechanisms need to be further explored. Otherwise, a number of limitations of the existing descriptive studies should be highlighted. These mainly pertain to differences in methodological approach (cross-sectional studies, small samples, outpatient/inpatient, and self-administered questionnaires versus semistructured interviews). Many confounding factors are insufficiently taken into account, such as age, sex, disease duration, and treatment with other psychoactive drugs. Indeed, in addition to antipsychotics, patients often consume anxiolytics, antidepressants, and other mood stabilizers with well-documented side effects of increased appetite and weight gain [72]. Finally, the impact of a bad socioeconomic environment, which frequently affects this vulnerable population, is rarely analyzed in studies. This socioeconomic insecurity is directly responsible for their limited access to medical care and healthy food and represents a major obstacle to the implementation of lifestyle and dietary rules, instead contributing to an unbalanced diet and insufficient physical exercise [6]. Moreover, the pharmacological treatment of EDs remains an underdeveloped field [73].

From a clinical point of view, we want to emphasize the importance of screening and assessment for comorbid EDs in patients presenting with schizophrenia. For example, antipsychotics have been implicated in binge eating symptomatology and other compulsive disorders [32, 33]. These compulsive symptoms may improve or cease when the antipsychotic is withdrawn or replaced. The clinician should distinguish this situation from a preexisting eating disorder. Interestingly, many screening tools for EDs exist, such as questionnaires or semistructured interviews, and should be included in these research protocols. Revised versions with a reduced number of items are now available. Their use within a multidisciplinary framework (physician, psychologist, and dietician) can allow a better approach to the EDs, leading to more accurate and reproducible assessment in monitoring. In addition, some of these self-rating scales have been translated and validated in several languages.

In conclusion, we should keep in mind that somatic and/or behavior comorbidities are often associated with schizophrenia. Given their frequency and negative impact on quality of life, the assessment of these specific EDs in schizophrenia needs to be improved. The screening and management of patients suffering from schizophrenia with EDs must be multidisciplinary. Coordinated actions among psychiatrists, general practitioners, psychotherapists, endocrinologists, nurses, dieticians and patients' families might be a valuable assistance to optimal care [74, 75]. Further studies should be performed in this domain because the available literature on the subject is very poor. The recommendations proposed for clinical practice are summarized in the following.

- Assess eating behaviors and screen for EDs, using validated tools if necessary.
- Recognize the somatic risks associated with EDs and assessing the patient's nutritional status (e.g., malnutrition, weight gain, and cardiometabolic disorders).
- Interview the patient about his or her quality and duration of sleep.
- Educate patients and family members on the side effects of antipsychotics (change in appetite, weight gain, risk of glucose intolerance, and lipid abnormalities).
- Adjust, if possible, the patient's antipsychotic treatment in accordance with his or her metabolic profile.
- Organize multidisciplinary and early management of EDs.
- Develop a personalized and comprehensive treatment strategy, including lifestyle measures (diet, adapted physical activity, and sleep hygiene).
- Use cognitive behavioral therapies that have proven effective in the field of EDs.

## Figures and Tables

**Table 1:** Data from descriptive studies evaluating the comorbidity of eating disorders in schizophrenia.

Authors	*N*	Sex (M/W)	Mean age (SD)	Assessment scale	Type of eating disorder	Prevalence (%)
Gotestam et al. 1995 [25]	10125	3544/6581	19–80	Staff-report questionnaire	AN, BN	**Men:** 0.81% for AN and 0.73% for BN **Women:** 4.01% for AN and 1.57% for BN
Striegel-Moore et al. 1999 [21]	22	22/0	Veterans	ICD	AN, BN	28%
Theisen et al. 2003 [32]	74	47/27	19.8 (±2.2)	QEWP	BED, BN	12.1% for BED 3.7% for BN
Stein et al. 2005 [22]	30	0/30	70 (±6.5)	EAT	AN	13.3%
Kluge et al. 2007 [33]	30	12/18	18–65	DSM-IV	BED	20%
Lundgren et al. 2010 [34]	68^*^	29/21	43.9 (±10.4)	NEQ, DSM IV, and QEWP	NES, BED	25% for NES 5.9% for BED
Palmese et al. 2011 [35]	100	39/61	46.5 (±10)	NEQ	NES	8%
M. H. Fawzi and M. M. Fawzi 2012 [12]	50	29/21	29.4 (±10.2)	EAT	Not determined	30%

ICD: International Classification of Diseases; QEWP: Questionnaire on Eating and Weight Patterns; EAT: Eating Attitude Test; DSM: Diagnostic and Statistical Manual of Mental Disorders; NEQ: Night Eating Questionnaire.

^*^Subjects characteristics: schizophrenia: 55.7%; bipolar disorder: 17.1%; major depressive disorder: 25.7%.
